# Transmission Risks of Schistosomiasis Japonica: Extraction from Back-propagation Artificial Neural Network and Logistic Regression Model

**DOI:** 10.1371/journal.pntd.0002123

**Published:** 2013-03-21

**Authors:** Jun-Fang Xu, Jing Xu, Shi-Zhu Li, Tia-Wu Jia, Xi-Bao Huang, Hua-Ming Zhang, Mei Chen, Guo-Jing Yang, Shu-Jing Gao, Qing-Yun Wang, Xiao-Nong Zhou

**Affiliations:** 1 National Institute of Parasitic Diseases, Chinese Center for Disease Control and Prevention, Shanghai, People's Republic of China; 2 WHO Collaborating Center for Malaria, Schistosomiasis and Filariasis; Laboratory of Parasite and Vector Biology, Ministry of Health, Shanghai, People's Republic of China; 3 Hubei Center for Disease Control and Prevention, Wuhan, Hubei, People's Republic of China; 4 Jiangling Institute of Schistosomiasis Control, Jiangling County, Hubei, People's Republic of China; 5 School of Public Health and Primary Care, The Jockey Club Chinese University of Hong Kong, Shatin, Hong Kong; 6 Jiangsu Institute of Parasitic Diseases, Wuxi, Jiangsu, People's Republic of China; 7 Normal University of Gannan, Ganzhou, Jiangxi, People's Republic of China; College of Public Health and Health Professions, United States of America

## Abstract

**Background:**

The transmission of schistosomiasis japonica in a local setting is still poorly understood in the lake regions of the People's Republic of China (P. R. China), and its transmission patterns are closely related to human, social and economic factors.

**Methodology/Principal Findings:**

We aimed to apply the integrated approach of artificial neural network (ANN) and logistic regression model in assessment of transmission risks of *Schistosoma japonicum* with epidemiological data collected from 2339 villagers from 1247 households in six villages of Jiangling County, P.R. China. By using the back-propagation (BP) of the ANN model, 16 factors out of 27 factors were screened, and the top five factors ranked by the absolute value of mean impact value (MIV) were mainly related to human behavior, *i.e.* integration of water contact history and infection history, family with past infection, history of water contact, infection history, and infection times. The top five factors screened by the logistic regression model were mainly related to the social economics, *i.e.* village level, economic conditions of family, age group, education level, and infection times. The risk of human infection with *S. japonicum* is higher in the population who are at age 15 or younger, or with lower education, or with the higher infection rate of the village, or with poor family, and in the population with more than one time to be infected.

**Conclusion/Significance:**

Both BP artificial neural network and logistic regression model established in a small scale suggested that individual behavior and socioeconomic status are the most important risk factors in the transmission of schistosomiasis japonica. It was reviewed that the young population (≤15) in higher-risk areas was the main target to be intervened for the disease transmission control.

## Author Summary

Schistosomiasis japonica is one of the serious infectious diseases causing problems in public health in People's Republic of China (P.R. China). The prevalence of schistosomiasis japonica is easily rebounding in the lake regions due to unique environmental settings. By considering the importance in assessing the risk factors for transmission of schistosomiasis, we conducted an epidemiologic investigation of schistosomiasis in Jiangling County, a lake region of P.R. China. Results showed that the top risk factors included integration of water contact history and infection history, infection times, main lifestyle of water contact, main recreation of water contact, etc., illustrated by both back-propagation artificial neural network and multivariable logistic regression approaches. It was found that the population at age 15 or younger having one or two times of infections was the high-risk group in the study settings. We concluded that socioeconomic factors are far more important than environmental factors in the transmission of schistosomiasis at local settings or in a small scale in Jiangling County, P.R. China.

## Introduction

In spite of schistosomiasis has been considered as one of neglected tropical diseases distributed in tropical and subtropical areas of the world, schistosomiasis ranks higher priority among four infectious diseases in alliance with HIV/AIDS, tuberculosis and hepatitis B in the People's Republic of China (P.R. China) [1]. As one unique disease among four major human schistosomiasis globally, schistosomiasis japonica caused by infection with *Schistosoma japonicum* is the most severe pathologically in infected humans [2]. It is also referred to as a “three factors disease” including parasites, snails and humans in accordance with its transmission circle [1]. Schistosomiasis japonica has been prevalent in the Yangtze River and the southward in P.R. China for more than 2100 years [3]. Although distinctive achievements have been gained in the national schistosomiasis control program with the efforts for more than 50 years in P.R. China [4], it is still facing the enormous challenges to prevent human from re-infections of *S. japonicum* affected by following facts [5], [6]. Firstly, the potential risks for resurgence of schistosomiasis japonica is still existed, particularly in the areas along the Yangtze River, such as Hunan, Hubei, Jiangxi and Anhui provinces, indicated by several reports that rebounding schistosomiasis occurred shortly after termination of the World Bank loan project on control of schistosomiasis in P.R. China in 2000 [7], [8]. Secondly, the human re-infection with *S. japonicum* is still a key issue for the disease transmission in those regions, and closely related to human social and economic status, consequently the epidemical extent of schistosomiasis varies from region to region [9]–[11]. Thirdly, it is noticed that current integrated strategies and measures of controlling schistosomiasis may not all suitable for certain areas [12], where major risk factors might be different from those areas where significant achievements had been achieved by integrated strategy [13]. Therefore, it is urgent to understand the different risk factors of *S. japonicum* transmission in different local settings.

It is well known that the complex relationship between risk factors and schistosomiasis transmission is existed, and is quite difficultly to be assessed by conventional statistical methods. Artificial neural network (ANN) can provide an encouraging computer-based method for recognition of transmission pattern due to its special features of nonlinearity, self-adaptation, and parallel processing [14]. ANN is composed of simple elements performing in parallel and being inspired by biological nervous systems, and a typical ANN includes three components, such as an input layer, a hidden layer, and an output layer. The back-propagation (BP) artificial neural network is one of ANN methods based on the standard back-propagation algorithm, which is most widely used in prediction, classification and characteristic recognition in the field of computer science and clinical diagnosis, but hard to be seen in the epidemiology study of tropical diseases [15]–[18]. Based on one of the major features of the ANN approach that the network exhibits a high degree of connectivity, of which the extent is determined by synaptic weights of the network, we put forward the hypnosis that the ANN model might able to illustrate the complex and nonlinear relationship between risk factors and intensity of schistosomiasis transmission through the risk ranking in the schistosomiasis transmission.

Therefore, our study aimed to identify risk factors for human infection with *S. japonicum* which varied to the socioeconomic and ecological factors, such as health-economic condition of family, human behaviors and habit of individual at a small scale, supported by integrated approach with BP artificial neural network and multivariate logistic regression model in order to offer guidelines for controlling the transmission of schistosomiasis japonica tailored to the local settings.

## Materials and Methods

### Study population and definition of variables

Six villages in Jiangling County, Hubei Province, P.R. China were sampled in random by the stratified clustered sampling, in which the prevalence and village was viewed as stratum and clustering, respectively. The study was performed from August to September in 2010 among local villagers with a range of age 6–60 from six villages as targeted population for the study. All of targeted villagers were screened by serum examination with indirect hemagglutination assay (IHA, LOT: 20100608 from Anji Pharmaceutical Science and Technology Co. Ltd.), then the positive individual with the titers of ≥20 in IHA was reconfirmed by stool Kato-Katz examination with three slides from a single stool specimen [19]. Individual data was collected including 18 items, such as gender, age, education level, infection history, primary infection, category of diseases, infection times, medical history (including treatment history, medicine varieties), history of water contact, main agricultural activities of water contact, main lifestyle of water contact and main recreation of water contact, frequency of water contact, months to contact with water, protective measures, sanitary condition (*i.e.* ground of courtyard and family latrines), and economic conditions of family, the results of IHA and Kato-Katz examination. The primary data of economic condition for each household were collected including following 3 aspects, including (i) electric appliance, e.g. electric rice-cooker, electric fan, TV set, refrigerator, etc., (ii) agricultural machine, e.g. tractor, cultivator, etc., and (iii) vehicles, e.g. bicycle, motorcycle, automobile, etc.. The economic status of each household was analyzed by principal component analysis and divided into 3 grades, *i.e.* good, general, and poor, according to the interquartile of principal component score. The specification of each variable in the study is listed in the Dataset S1 and Dataset S2.

### Statistical analysis

Crude associations between infection and each of the variables considered for the analysis were analyzed by chi-square test. The important risk-factors were selected through fitting multivariate logistic regression model in three steps [20]. Firstly, the variables selection was performed by a univariable analysis of each variable. Secondly, we employed variables based on results from the univariable analyses to develop model through forward stepwise regression. Thirdly, following the fit of multivariable, the importance of each variable included in the model was verified, and those variables that do not contribute to the model were eliminated. The new model was evaluated by fitness analysis, and comparison was undertaken between the new and the old models.

The significance of risk factors was estimated by standard partial regression coefficients and odds ratios. The logistic regression model was established employing seropositive rate that is a result of human exposure to the risks as the dependent variable with 26 independent variables, such as prevalence level (prevalence), village level (village), ground of courtyard (courtyard), source of drink water (drink water), family latrines (latrines), distance to site of positive snail (distance), the family with past infection (past-infection), economic conditions of family (economic conditions), age group (age), gender, education level (education), marriage, occupation, infection history, time interval from the first infection to now (time interval), category of the diseases (category), treatment history (treatment), medicine varieties (medicine), infection times, history of water contact (infested water), main agricultural activity of water contact (agriculture activity), main lifestyle of water contact (main lifestyle), main recreation of water contact (main recreation), frequency of water contact (frequency), months to contact with water (months), protective measures (measure), and possible interaction between independent variables. The fitness of the logistic regression models were tested by the summary measures based on the likelihood chi-square statistics. Akaike's information criterion (AIC) and Schwartz criterion (SC) were used to define the optimal models. The smaller the values of both AIC and SC were, the better the model is. The determination of coefficient (R^2^) presents the interpretation of the model for independent variable, when R^2^ is close to zero it is indicated that there is no relationship between the dependent and the independent variables in the models. A model was considered relatively parsimonious when the fitting indices changed little [20], [21]. Statistical procedures were carried out using the SAS9.1.3 computer packages (Statistical Analysis System, RTI, Cary, North Carolina, USA).

### BP artificial neural networks analysis

All of the data collected from the field were employed into the BP artificial neural network [22] (Figure S1). Four steps were processed in selection of the risk factors of *S. japonicum* infection to construct the BP artificial neural network [23], [24]. In the first step, an initial network was constructed with an input layer, one hidden layer, and an output layer, on the basis of heuristic information. In the initial network, the input layer included 26 neurons (or independent variables) and possible interaction between independent variables (Dataset S1); the node numbers of hidden layer were determined based on the formula of ***l***<***n***-1, where ***l*** is the number of the nodes in the hidden layer, and n is the number of nodes in the input layer, and then followed by the trial and error method to identify the best numbers of the node; and the output layer was set into one node, which was the result of serum test indicating the exposure level to the transmission risks. The transfer function for hidden layer is tansig and that for the output layer is purelin. In the second step, the weight values were initialized firstly, with its function of taking a network object as input and returns a network object with all weights and biases initialized. Then after the network weights and biases were initialized, the network was ready for training based on gradient-based method. The process of training a neural network involved tuning the values of the weights and biases of the network to optimize network performance, of which the procedure was automatically accomplished by corresponding functions, *i.e.* the network training function. It then evaluated by the network performance function or mean square error (MSE) that measures the performance of network. The most appropriate network was determined empirically during the training process that is a black box process. After a large number of applications of input neurons, the network converged to a solution that minimized the least square difference between the expected and measured outputs. With the magnitude of the gradient became very small, the training reached a minimum of the performance. In the third step, when the multilayer network was trained, data were automatically divided into training (70%), validation (15%) and test (15%) sets, in order to perform the cross-validation of the network and to evaluate the ability of a trained network. The error on the validation set was monitored during training until the training stopped when the validation increased over a given iterations. The test errors that used in the comparison of different models were set and plotted during the training process. If the test error reached a minimum at a significantly different iteration number than the validation set error, it might hint a poor division of the data set. The correlation coefficient (R) was used to assess the relationship between the outputs of network and the targets. In the forth step, the salient variables were selected according to mean impact value (MIV) which was an index reflecting changes of weights matrix in neural networks after the construction of the most appropriate ANN [25], [26]. The size of MIV's absolute value reflects the importance of corresponding variables for dependent variable and its sign denoted the direction of correlation with dependent variable (Figure S2). The positive values indicate a direct relationship to the output variables, and the negative values reflect an inverse relationship. Values near zero reviewed that the input variable had little or no relationship to the output variables. Then, we re-constructed a new network after the variable with the smallest was deleted from the most appropriate ANN.

We repeated the above four-step process until the optimal network could be found. BP artificial neural network was developed in the Matlab (Matrix Laboratory, Math Works Company, USA, R2011a software).

### Ethical statement

Ethical clearance had been granted by the Ethics Committee of the National Institute of Parasitic Diseases, Chinese Center for Disease Control and Prevention in Shanghai, P.R. China (Ref No: 20100802-1). The objectives, procedures and potential risks were orally explained and informed to all participants through village head. All adult participants gave written, informed consent, and child participants provided written informed consent signed by their parents or guardians before participating in this study, of which the research protocol was also approved by Institutional Review Board.

## Results

### Prevalence data

The study was conducted among 2339 villagers from 1247 families in the six villages, namely Sanzha, Qinggang, Zhongqiao, Luyangtai, Liugang, and Qingan of Jiangling County. Among which 448, 411, 377, 311, 248 and 544 villagers were from above 6 villages, respectively, including 1155 (49.38%) women and 1184 (50.62%) men. The median of age was 47 years, and interquartile range was from 38 to 55 years. The population was composed of 6 age-groups, such as ≤15 years old (8.25%), 15–25 (6.11%), 25–35 (6.15%), 35–45 (25.14%), 45–55 (30.10%), >55 (24.24%). Their occupation consisted of 1843 (78.79%) from farmers, 253 (10.82%) from children and students, and 243 (10.39%) individuals in other occupation. The positive rate of IHA was 15.09% (353/2339), varied from 5.88% to 27.32% at village level (Table 1).

**Table 1:** The results of IHA and Kato-Katz in residents of study villages.

	IHA results			Kato-Katz results			
village	Total	Number of positive	Positive rate (%)	Total	Number of positive	Positive rate (%)	Adjusted infection rate (%)
Sanzha	448	84	18.75	70	12	17.14	3.21
Qinggang	411	70	17.03	66	10	15.15	2.58
Zhongqiao	377	103	27.32	90	10	11.11	3.04
Luyangtai	311	44	14.15	40	7	17.50	2.48
Liugang	248	20	8.06	18	1	5.56	0.45
Qingan	544	32	5.88	30	1	3.33	0.20

### Statistical analysis

The results from crude association analysis showed that the variables to associate with infection, or the results of serum examination, were in turn into 20 risk factors investigated as follows: prevalence, village, courtyard, latrines, past-infection, economic conditions, education, infection history, category, treatment, medicine, infection times, infested water, agriculture activity, main lifestyle, main recreation, frequency, measure, months, and integration (Table 2).

**Table 2:** The results of logistic regression model and BP artificial neural network.

Original variable	Crude Association Analysis		Logistic Regression Model					BP ANN	
	χ^2^	P Value	variables/dummy variables	Standard partial regression coefficient	Odds Ratio (OR)	95%CI		mean impact value (MIV)	Rank of |MIV|
						lower	upper		
Prevalence level (PL)	68.5673	<0.0001	-	-	-	-	-	0.0054	10
Village level (VL)	95.6636	<0.0001	-	-	-	-	-	−0.0032	12
			Sanzha	0.1200	1.7380	1.205	2.507	-	-
			Qinggang	0.1052	1.6510	1.117	2.440	-	-
			Zhongqiao	0.2008	2.6920	1.868	3.880	-	-
			Luyangtai	0.0787^b^	1.5220	0.992	2.336	-	-
			Liugang	−0.045^a^	0.7670	0.446	1.319	-	-
			Qingan	-	1.0000	-	-	-	-
Ground of courtyard (GC)	11.6019	0.0206	-	-	-	-	-	−0.0053	11
Family latrines	16.8748	0.0007	-	-	-	-	-	0.0028	13
(FL)Family with past infection (FPI)	949.524	<0.0001	-	-	-	-	-	0.0231	2
Economic conditions of family (ECF)	76.6435	<0.0001	-	-	-	-	-	0.0020	15
			Good	−0.3280	0.2660	0.191	0.372	-	-
			General	−0.2822	0.3590	0.277	0.466	-	-
			Poor	-	1.0000	-	-	-	-
Age group (AG)	1.1177	0.9525						−0.0110	6
			>55	−0.4060	0.1790	0.110	0.292	-	-
			45–55	−0.3831	0.2200	0.137	0.352	-	-
			35–45	−0.3134	0.2700	0.168	0.434	-	-
			25–35	−0.1240	0.3920	0.215	0.715	-	-
			15–25	−0.1150	0.4190	0.229	0.766	-	-
			≤15	-	1.0000	-	-	-	-
Education level (EL)	9.5793	0.0481	Literacy/Illiteracy	−0.1933	0.4530	0.355	0.578	−0.0059	8
Infection history (IH)	24.1748	<0.0001	-	-	-	-	-	0.0145	4
Category of the disease (CD)	25.6729	<0.0001	-	-	-	-	-	−0.0061	7
treatment history TH)	35.3426	<0.0001						-	-
Medicine varieties (MV)	47.7618	<0.0001	-	-	-	-	-	−0.0057	9
Infection times (IT)	38.2776	<0.0001	-	-	-	-	-	−0.0110	5
			>10 times	0.1103	1.5110	1.074	2.126	-	-
			6–10 times	0.1550	1.9870	1.352	2.920	-	-
			3–5 times	0.0880	2.0760	1.166	3.698	-	-
			1–2 times	0.1212	3.9700	2.043	7.716	-	-
			0 times		1.0000	-	-	-	-
History of water contact (HWC)	12.2687	0.0005	Yes/No	0.033^a^	1.1750	0.792	1.743	−0.0152	3
Main agriculture activity of water contact (AAWC)	14.2606	0.014	-	-	-	-	-	-	
Main lifestyle of water contact (LSWC)	17.9678	0.003	-	-	-	-	-	−0.0024	14
Main recreation of water contact (RWC)	13.3443	0.0097	-	-	-	-	-	−0.0017	16
Frequency to contact with infested water (FCW))	19.1222	0.0018	-	-	-	-	-	-	-
Protective measure(PM)	14.2109	0.0008	-	-	-	-	-	-	-
Months to contact with water (MCW)	19.6447	0.0006						-	-
Integration of water contact history and infection history (WCH-IH)	28.0919	<0.0001	-	-	-	-	-	0.0305	1

Note: a:p>0.05, b:0.05<p,0.06.

The stratified analysis, namely Cochran Mantel Haenszel (CMH), was used to determine the correlation between infection history, infested water and IHA positive rate, in which infection history was viewed as stratification factor. The results of CMH showed that OR_infection history = 0_ was 1.0426 (95%CI 1.0019–1.0850), OR _infection history = 1_ was 0.7198 (95%CI 0.7198–0.5051), and the ORs in the two strata was significant difference justified by the Breslow-Day test, with its total odds ratio of the two strata (OR_CMH_) was 1.4510 (95%CI 1.1809–2.365) and common odds ratio (OR_common_) was 1.8969 (95%CI 1.3189–2.7283).

The logistic regression model was established in a good fitness which was shown by following three values (Table 3), *i.e.* the AIC was 1793.805, R^2^ was 0.5984, and test of Likelihood ratio (χ^2^) was 1358.1638 (P<0.0001). There were six independent variables in the logistic regression, which were in turn village, economic conditions, age, education, infection times, infested water (Table 2). The OR values were calculated in the following five aspects: including (i) the positive rate of IHA was lower among population elder when age 15 or younger as reference, the OR values of 55, 45–55, 35–45, 25–35, and 15–25 age group were in turn 0.1790, 0.22, 0.27, 0.392, 0.419, respectively; (ii) the OR value of higher education was 0.4533 when illiteracy as reference; (iii) the OR values of infection rate in Sanzha, Qinggang, and Zhongqiao were 1.738, 1.6510, and 2.692, respectively, when the infection rate of Qingan as reference; (iv) the OR values of good family and general family were 0.266 and 0.3590, respectively, when poor family as reference; (v) the OR value of the IHA positive rate was 3.9700, highest among population with 1 or 2 infection times when 0 times as reference (Table 2).

**Table 3:** The fitness index of logistic regression and BP artificial neural network.

Model	Index	Value
Logistic regression	Akaike's information criterion	1793.805
	test of Likelihood ratio	1358.1638 (p<0.0001)
	the determination coefficient	0.5984
Artificial Neural Network	mean squared error	0.0734
	the magnitude of the gradient	0.0019082
	Validation checks	0
	correlation coefficient	0.65361

### BP artificial neural networks analysis

**Figure 1:**
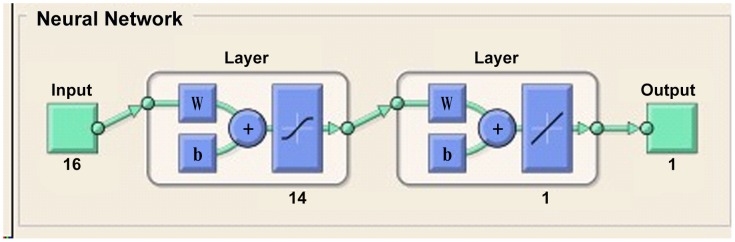
The configuration of the final BP ANN.

**Figure 2:**
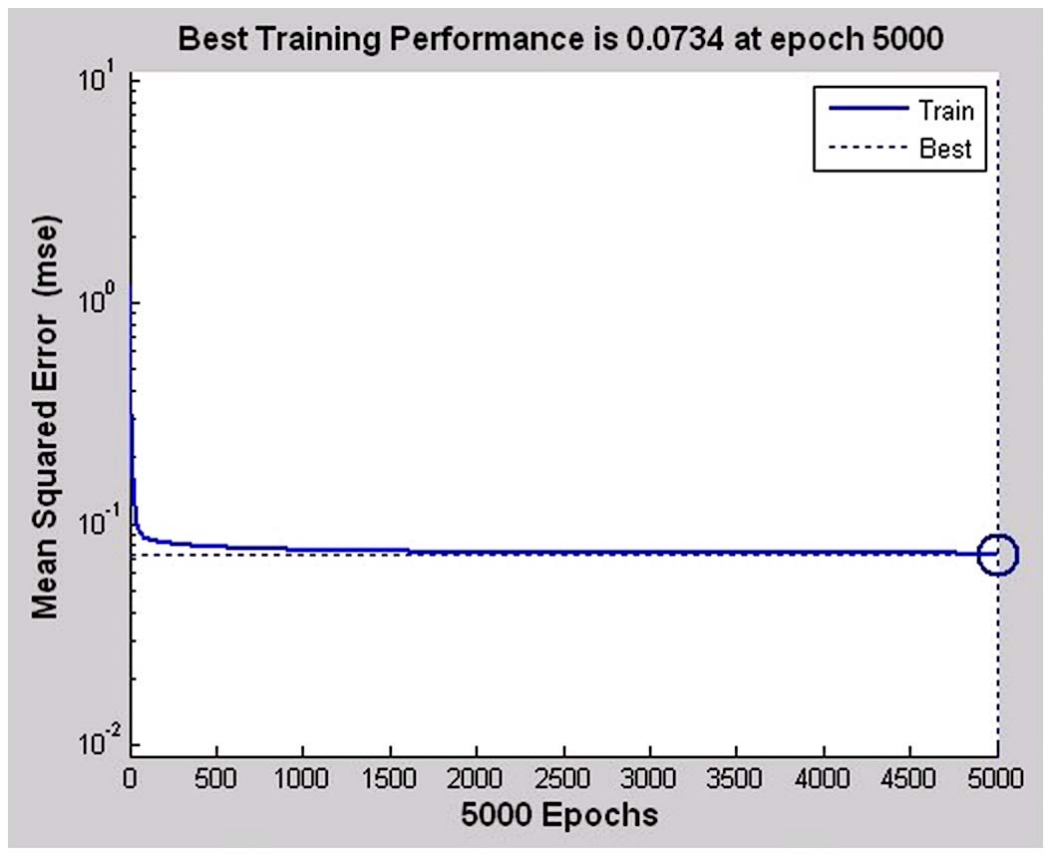
The plot of error mean squared in training the ANN. This plot shows the process of training the ANN involves tuning the values of the weights and biases of the network to optimize network performance. The mean squared error is the default performance function for feed-forward – the average squared error between the network outputs and the target.

**Figure 3:**
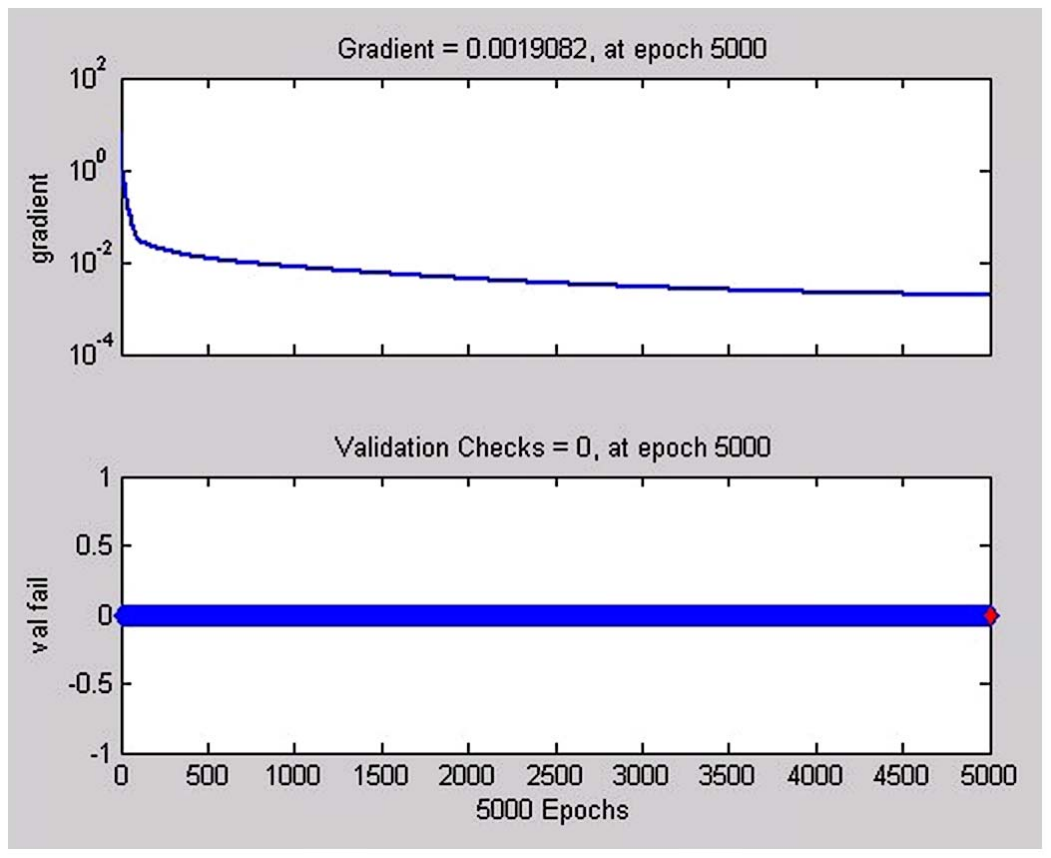
The plots of the model performance function, through the magnitude of the gradient (upper), and the number of validation checks (down). After the training was complete, the network performance would be checked to determine if any changes need to be made to the training process, the network construction or the data sets. This plot is reflecting the performance progress with the value of the performance function and the magnitude of the gradient, showing the gradient will become very small as the training reaches a minimum of the performance.

**Figure 4:**
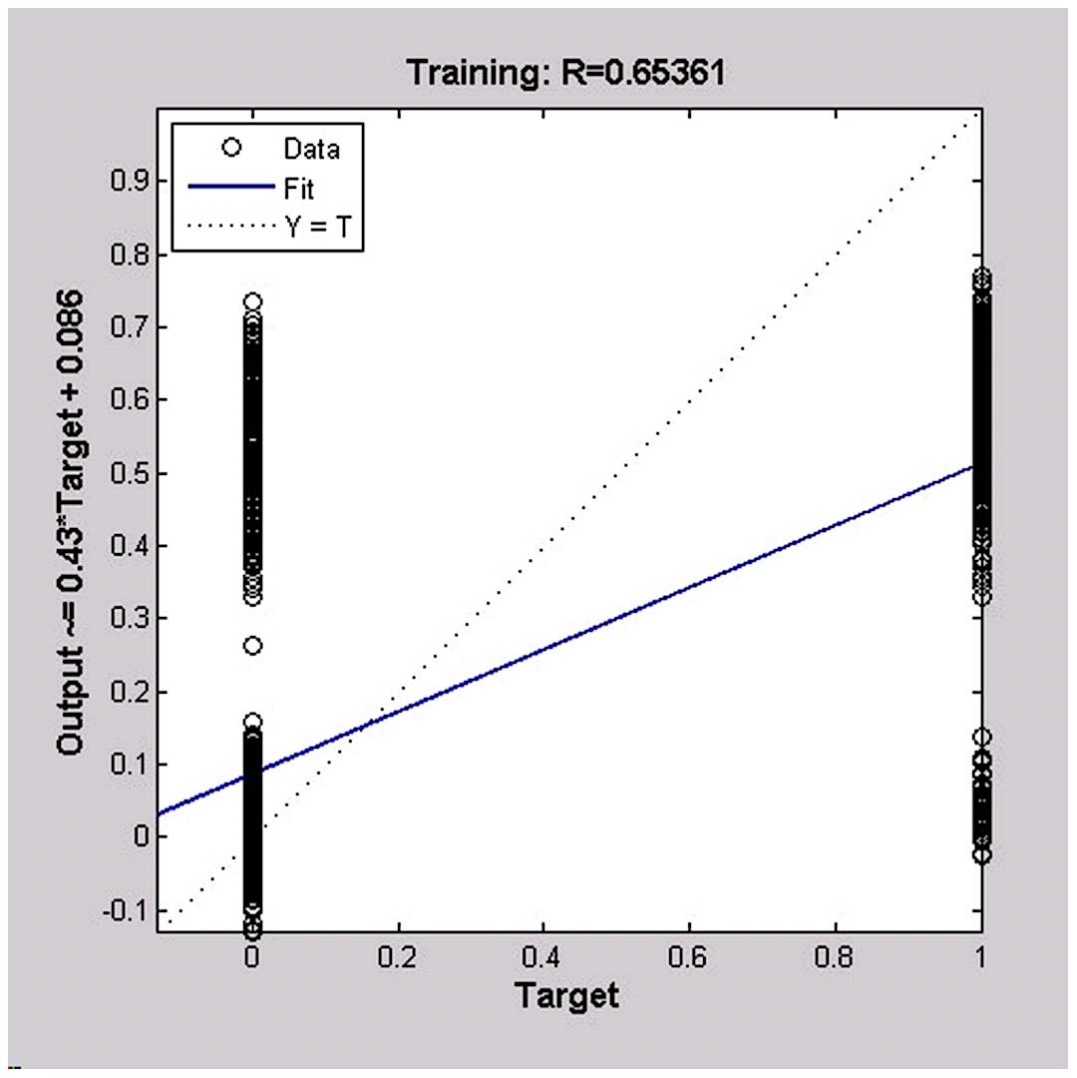
The regression plot to validate the network trained. The regression plot showed the relationship between the outputs of the network and the targets, showing the training of the network were perfect, or network outputs were exactly equal to the targets, but the relationship is rarely perfect in practice.

The final established BP artificial neural network consisted of one input layer with 16 neurons, one hidden layer with 14 nodes, and one output layer with 1 neuron. The good fitness of the final network was well performed indicated by following four values (Table 3), such as 0.0734 for MSE, 0.0019082 for the magnitude of the gradient, 0 for the number of validation checks, and 0.65361 for R (Figure 1–4). The network performance was in a good pattern when the network contained 16 risk factors (Table 2). According to the absolute value of MIV, the 16 most important risk factors in the rank were in turn as follows: integration (MIV = 0.0305), past-infection (MIV = 0.0231), infested water (MIV = −0.0152), infection history (MIV = 0.0145), infection times (MIV = −0.110), age (MIV = −0.0110), category(MIV = −0.0061), education (MIV = −0.0059), medicine (MIV = −0.0057), prevalence (MIV = 0.0054), courtyard (MIV = −0.0053), village (MIV = −0.0032), latrines (MIV = 0.0028), main lifestyle (MIV = −0.0024), economic conditions (MIV = 0.0020), and main recreation (MIV = −0.0017) (Table 2).

## Discussion

To our knowledge, it is the first time to apply the BP artificial neural network in the neglected tropical diseases, with its specific purpose to extract the important risk factors that contributed to the disease transmission, and to guide the interventions. The major reason we use this approach is that BP artificial neural network is able to improve our understanding the risk factors in transmission of *S. japonicum* with nonlinear patterns, which reflected by following three features, *i.e.* the model of each neuron in the network includes a nonlinear activation function, the network contains one or more layers that are hidden from both the input and output nodes, and the network exhibits a high degree of connectivity, the extent of which is determined by synaptic weights of the networks. Hence, our investigation was undertaken by two parts, the first part is the design, evaluation, and interpretation of BP artificial neural network, and the second part is the extraction of risk factors contributed to the transmission of *S. japonicum* in Jiangling local settings in comparison with the results both from BP artificial neural network and from the conventional statistical model, or logistic regression model.

In the first study part, the establishment of ANN was performed. The IHA results, that can detect both current and past infections, was used as a dependent variable to explore the risk factors for human exposure to the risk environment, since the serological data along with epidemiological data can reflect the real status in the prevalence and transmission of schistosomiasis [27]–[29]. We presented the Kato-Katz examination results in Table 1, but were not included in the analysis due to following three reasons. Firstly, Kato-Katz technique is plagued by lessened sensitivity in individuals when the worm burden is low [30]. Secondly, inter-individual variation in egg production, as well as repeated egg counts on one individual varied considerably [31], as a result of many infected individuals remain undetected. Thirdly, the compliance of villagers to provide stool specimens were low in those areas where intervention has been under taken for a long period, in particular Jiangling County [32]–[34], where only 314 out of 353 seropositive individuals to offer stool example in the study (Table 1).

In this study, we considered the interaction between infection history and infested water in view of following two reasons. The first reason is that the individual behavior in daily life which we observed was mainly related to both infection history and infested water. The second is that the stability of model would be fluctuated if interaction among all of variables was considered. This phenomenon is also supported by the results of the stratified analysis showing an interaction between infection history and infested water. But, we found that the product term, which referred only to interaction on a multiplication scale, was no significant in the logistic models after the product term was added into the logistic models. Due to logistic regression model is suitable for multiplicative model [35], we created a new variable, namely integration (Dataset S1), to be included into the network study.

The BP artificial neural network was established in the study by taking the advantages of the network model, *e.g.* the relationship between schistosomiasis and its risk-factors is believed to be non-linear which more suitable for using ANN approach [36]. Generally, the input variables of the network were selected on the basis of professional knowledge and experience, since the precision of the network will be lowered if the network contains some variables which were less significant. A proper neural network was synthetically evaluated by the following four indices: MSE, the magnitude of gradient, validation checks, and correlation coefficient R. The input variables were screened according to the MIV of variables in the networks. We constructed the different networks with 27, 26, 25, …, 13 input variables respectively, and found that the differences between MIVs of input variables in networks gradually decreased with the input variables deleted. There were little difference among MIVs of input variables when the networks contained less than or equal to 16 input variables. However, the performance of the networks with less than 16 input variables was worse than one of the networks with 16 input variables. Finally, we decided that the network with 16 input variables was the optimal network based on the performance of BP ANN and MIV of variables in the networks (Text S1, Table 2, and Figure S3). Among the input variables, integration was introduced into the ANN, which could help us to take more advantages in understanding the complexity of schistosomiasis transmission. Since this interaction is taking the leading role in the transmission of the disease, and only the most important interaction term could be introduced into the model according to the principle of frugality, otherwise the stability of model would be poor if more interaction entered into the network.

Integration was the most important factor for *S. japonicum* infection according to the ranking absolute value of MIV in the BP artificial neural network, and followed by the infested water and infection history. Therefore, the top three risk factors were directly related to past-infection, which indicated that the health education and health improvement have to be considered in the implementation of the intervention locally. Meanwhile, we also found the other factors, such as courtyard, latrines, economic conditions, were closely related to transmission level of schistosomiasis. So that human *S. japonicum* infections appeared family clustering in the study areas, probably due to the higher intensity of schistosomiasis which influenced by both genetic heterogeneity and acquired immunity [37], [38]. Future studies on combining biological factors with socio-economic factors are recommended to understand the family cluster of infections. The risk factors, ranking from fifth to ninth, were in turn infection times, age, category, education, and medicine, which more relevant to the infection history. Hence, the higher IHA positive rate of population is prone to be infected with *S. japonicum*. In this study, prevalence was introduced into the network as a confounding factor, in the light of ranking the absolute value of MIV of prevalence and village which was listed in the tenth and twelfth, respectively. So that we are able to get conclusion that the transmission of schistosomiasis is not only influenced by the prevalence degree of each village, but also other factors at the village level, such as habitats of snail intermediate host. For instance, the mean density of infected snail in Zhongqiao village was highest among the six study villages based on the annual reports in 2010. Therefore, the integrate intervention in combination with behavior intervention as well as snail control is recommended to be taken for the higher risk villages where density of infected snails is higher [39].

In the second study part, an agreement between the BP artificial neural network and the logistic regression model was obtained. For example, R^2^ of the logistic regression model and R from ANN cued that transmission of schistosomiasis was both influenced by factors related to the socioeconomic and environmental factors [8], [40]–[42]. Both MIV from the BP artificial neural network and the standard partial regression coefficient from the logistic regression model indicated that history of infection and history of water contact were the main risk factors at the village level. Therefore, we concluded that socioeconomic factors were far more important than environmental factors in the transmission of schistosomiasis at local settings of Jiangling County, rather than the distance to infested water which were explored in previous studies [9]. In addition, the result of both methods showed that occupation of participants was not related to their infection of *S. japonicum*, it might be because the farmers was dominated accounting for 78.79%, while fisherman and boatman who were viewed as the high risk population accounted only for 0.38% and 0.73%, respectively, in the study population [43]. As a result, there was no any difference in exposure experience to the infested water among occupations.

The results from logistic regression can epidemiologically explain the contribution of each variable showed in their OR and regression coefficient R at the significance level of the model. We found in the study that five variables including village, economic conditions, age, education, infection times were significantly associated with the IHA positive rate. Although infested water was not significantly associated with the IHA positive rate, the fitting of the model became poor after it was rejected from the model. All above facts indicated that water contact due to poor behavior was important factors to the transmission of schistosomiasis. While the results based on the OR among different age groups from the logistic regression model indicated the younger the local residents were, the higher risk to be infected with *S. japonicum* they had, which in consistence with the results of previous studies [40], [44], [45]. The study region in Jiangling County was still a hyper-prevalent area of *S. japonicum* in accordance with the current national classification of prevalence, indicating the key objects for the control programme will be the population at age 15 or younger in local settings. The economic status of family and educational level influenced in some extent on the transmission of *S. japonicum*, which suggested that speeding up local economic development and raising average educational level would promote effectiveness of controlling the transmission of schistosomiasis.

We applied both ANN and logistic regression approaches in the study, because ANN is data-driven self-adaptive methods in that there are a few priori assumptions about the models for problems under study. ANN can be viewed as one of the multivariate nonlinear nonparametric statistical methods [5], and has more general and flexible functional forms than the traditional statistic methods can effectively deal with. It has been shown that a network can approximate any continuous function to any desired accuracy [6]. So, all variables were simultaneously introduced into the network. A great difference from ANN approach, logistic regression is difficult to explore the multi-colinearity which is a common phenomenon in schistosome infections. We screened the variables through the forward stepwise regression in logistic regression which can easily to highlight the risk population. Although ANN has many advantages be incomparable with traditional statistics, there are some difficulties that have not yet been solved, for instance, how to determine the numbers of the node in the hidden layer. In the study, the best numbers of the node, for instance 34 nodes in our study, in the hidden layer are identified on the basis of the formula, *l*<*n*-1, relevant to the numbers of the nodes in the hidden layer and the numbers of nodes in the input layer, as well as the trial and error method.

All in all, our study found that family clustering was the feature of prevalence of schistosomiasis japonica in the study area. The population at age 15 or younger was focal population in the schistosomiasis intervention in the area. Individual behavior factors were main risk factors of schistosomiasis transmission in the small scale. Consequently, strategy of controlling the transmission of schistosomiasis japonica should differ from population to population and from area to area [46]. For example, healthy education and intervention need to focus on children and students, and protective measure are put an emphasis on adults. One of the advantages of ANN is non-linearity what we found is more suitable to explore the relationship between schistosomiasis transmission patterns and its risk factors that believed to be non-linear [36]. The results from the investigation further demonstrated that BP artificial neural network is better than that of the conventional linear regression model in terms of capturing the nonlinear relationships with greater accuracy [47]. The integrated methods of combining BP artificial neural network and conventional statistical model are benefit for us further understanding the epidemiological characteristics of schistosomiasis which will provide sound information for surveillance and response [48].

One of the limitations in the study is that not all risk factors were considered as its entirety in the analysis, e.g. some of ecological and meteorological elements, which may lost some of information in our study. Furthermore, BP artificial neural network technology itself is continuously developing, and algorithm of network is gradually improved [49]. Therefore, it is promising for the application of BP artificial neural network to the epidemiological study in neglected tropical diseases, and to understand risk factors of disease transmission.

## Supporting Information

Checklist S1 **STROBE Statement.**
(DOC)

Dataset S1 **The specification of variable assignment.**
(DOC)

Dataset S2 **The variable assignment of household economic conditions.**
(DOC)

Figure S1 **The initial BP ANN includes an input layer, one hidden layer, and an output layer.** In the form of ‘neural activity’, the first hidden layer is fed forward from the input layer; then its resulting outputs are in turn applied to the second hidden layer; and so on for the rest of the network, and the error is back-propagated (layer by layer) to modify the weights of the connections.
(TIF)

Figure S2 **The flowchart to select variables on the basis of MIV.**
(TIF)

Figure S3 **The plot showing the performance of network which containing different number of risk factors.**
(TIF)

Text S1 **The procedure of screening risk factors.**
(DOC)
