# Supplementary material for: Transmission Risks of Schistosomiasis Japonica: Extraction from Back-propagation Artificial Neural Network and Logistic Regression Model
Source: PLoS Negl Trop Dis. 2013 Mar 21;7(3):e2123. doi: 10.1371/journal.pntd.0002123 (PMC3605232; doi:10.1371/journal.pntd.0002123)
Supplement: Dataset S1 — The specification of variable assignment. (DOC) [file pntd.0002123.s002.doc]

**Supplementary Dataset S1**

**Dataset S1** The specification of variable assignment

| Variable (shorthand) | Specification | Remarks |
| --- | --- | --- |
| Prevalence level (prevalence) | It means stratum information of stratified clustered sampling method. It was divided into three levels according to Jiangling County’s annual report in 2009: 1=’<1%’, 2=’2%~1%’, 3=’>=2%’. | Input Variable |
| Village level (village) | It means village sampled in this study: 1=Qingan, 2=Liugang, 3=Luyangtai, 4=Zhongqiao, 5=Qinggang, 6=Sanzha | Input Variable |
| Ground of courtyard (courtyard) | It means courtyard ground of household to reflect the sanitary conditions, and it is divided into 5 categories: 0=Without, 1=Soil, 2=Sand and stones, 3=Cement, 4=Others | Input Variable |
| Source of drink water (drink water) | It means source of drink water in the household to reflect the sanitary conditions, and it is divided into 6 categories: 1=Piped water, 2=Well water, 3=River, 4=Ditch water, 5=Lake, 6=Others | Input Variable |
| family latrines (latrines) | It means latrine used in the household to reflect the sanitary conditions, and it is divided into 4 categories: 1=Stercorary, 2=Marsh gas tank, 3=Three-cell latrine, 4=Water closet | Input Variable |
| Distance to site of positive snail (distance) | It means distance from residence to positive snail point, and it is divided into the 5 levels: 1='<=100m', 2='100-200m', 3='200-500m', 4='500-1000m', 5='>1000m' | Input Variable |
| the family with past infection (past-infection) | It means whether there was case inflected with *Schistosoma japonicum* in the past, it is a binary variable: 0=Without, 1=With | Input Variable |
| Economic conditions of family (economic conditions) | It is a variable to reflect economic conditions of household. 1=’<25%’, 2=25-75%, 3=’>=75%’ | Input Variable |
| Age group (age) | 1='<15', 2='15-25', 3=25-35', 4='35-45', 5='45-55', 6='>='55' | Input Variable |
| Gender (gender) | 0=Female, 1=Male | Input Variable |
| Education level (education) | 0=Illiteracy, 1=Primary school, 2=Middle school, 3=High school, 4= Junior college and others | Input Variable |
| Marriage (marriage) | 1=Single, 2=Married, 3=Divorced, 4=Widower or widow | Input Variable |
| Occupation (occupation) | 1=Farmer, 2= Preschool child and student, 3= Others | Input Variable |
| Infection history (infection history) | 0=No, 1=Yes | Input Variable |
| Time interval from the first infection to now (time interval) | 0=Without infection history, 1= '<= 3 years', 2= '>3 years' | Input Variable |
| Category of diseases (category) | It means the status of infection with schistosome in the recent period, is divided into the 4 categories: 0=Without infection history, 1=Chronic, 2=Acute, 3=Terminal | Input Variable |
| Treatment history (treatment) | 0=Without infection history, 1='0 times' 2='1-2 times', 3='3-5 times', 4='>=5 times' | Input Variable |
| Medicine varieties (medicine) | 1=PAT, 2=Nithiocyamine, 3=PZQ, 4=PAT and Nithiocyamine, 5=Nithiocyamine and PZQ, 6= All of three | Input Variable |
| Infection times (infection times) | 0=Without infection history, 1='1-2 times', 2='3-5 times', 3='>=5 times' | Input Variable |
| History of water contact (infested water) | 0=No, 1=Yes | Input Variable |
| Main agricultural activity of water contact (agricultural activity) | 0=Without contact history, 1=Plowing, 2=Fishing, 3=Cutting water plants, 4=Grazing | Input Variable |
| Main lifestyle of water contact (main lifestyle) | 0=Without contact history, 1=Washing clothes, 2=Washing vegetables, 3=Washing farm implement, 4=Wading, 5=others | Input Variable |
| Main recreation of water contact (main recreation) | 0=Without contact history, 1=Paddling, 2=Swimming, 3=Fishing, 4=Others | Input Variable |
| Frequency to contact with infested water (frequency) | 0=Without contact history, 1=' >=200 days', 2='200-150 days', 3='150-100 days, 4='100-50 days', 5=' <50 days' | Input Variable |
| Months to contact with water (month) | 0=Without contact history, 1=Feb~Mar, 2=Apr~May, 3=June~July, 4=Aug~Oct | Input Variable |
| Protective measure (measures) | 0=Without contact history, 1=Without protective measures, 2=With protective measures | Input Variable |
| integration of water contact history and infection history (integration) | 0=None of both, 1= With infection history only, 2=With contact history of infested water, 3= Both | Input Variable |
| Blood examination (blood examination) | 0=Negative, 1=Positive | Output Variable |
