# Supplementary material for: Transmission Risks of Schistosomiasis Japonica: Extraction from Back-propagation Artificial Neural Network and Logistic Regression Model
Source: PLoS Negl Trop Dis. 2013 Mar 21;7(3):e2123. doi: 10.1371/journal.pntd.0002123 (PMC3605232; doi:10.1371/journal.pntd.0002123)
Supplement: Dataset S2 — The variable assignment of household economic conditions. (DOC) [file pntd.0002123.s003.doc]

**Supplementary Dataset S2**

**Dataset S2** The variable assignment of household economic conditions

| Variable Name | Specification |  | Variable Name | Specification |
| --- | --- | --- | --- | --- |
| house | 0=No, 1=Yes |  | motor | 0=No, 1=Yes |
| phone | 0=No, 1=Yes |  | tractor | 0=No, 1=Yes |
| TV | 0=No, 1=Yes |  | thresher | 0=No, 1=Yes |
| VCD | 0=No, 1=Yes |  | till | 0=No, 1=Yes |
| fan | 0=No, 1=Yes |  | machine | 0=No, 1=Yes |
| pan | 0=No, 1=Yes |  | cattle | 0=No, 1=Yes |
| washing | 0=No, 1=Yes |  | field | 0=No, 1=Dry field, 2=Paddy field, 3=Dry and paddy field |
| shrink | 0=No, 1=Yes |  | earning sources | 1=Crop, 2=work outside the home, 3=others |
| fridge | 0=No, 1=Yes |  | expenses | 1=in debt, 2=balance, 3=in credit |
| aircondition | 0=No, 1=Yes |  | income and expenditure | 1=production, 2=life, 3=medical |
| bicycle | 0=No, 1=Yes |  |  |  |
