# Supplementary figures and images for: Transmission Risks of Schistosomiasis Japonica: Extraction from Back-propagation Artificial Neural Network and Logistic Regression Model
Source: PLoS Negl Trop Dis. 2013 Mar 21;7(3):e2123. doi: 10.1371/journal.pntd.0002123 (PMC3605232; doi:10.1371/journal.pntd.0002123)

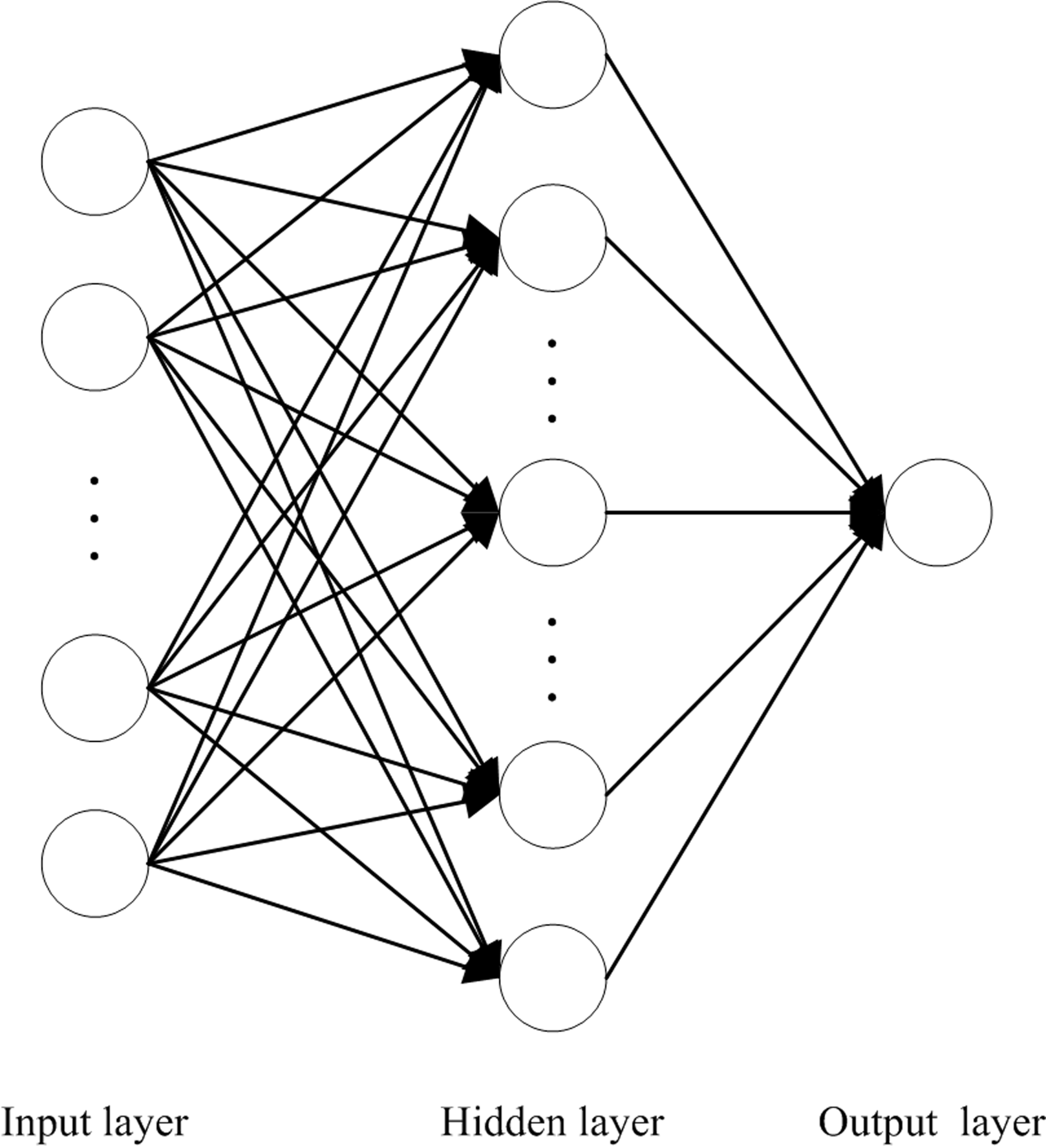

Supplement: Figure S1 — The initial BP ANN includes an input layer, one hidden layer, and an output layer. In the form of ‘neural activity’, the first hidden layer is fed forward from the input layer; then its resulting outputs are in turn applied to the second hidden layer; and so on for the rest of the network, and the error is back-propagated (layer by layer) to modify the weights of the connections. (TIF) [file pntd.0002123.s004.tif]

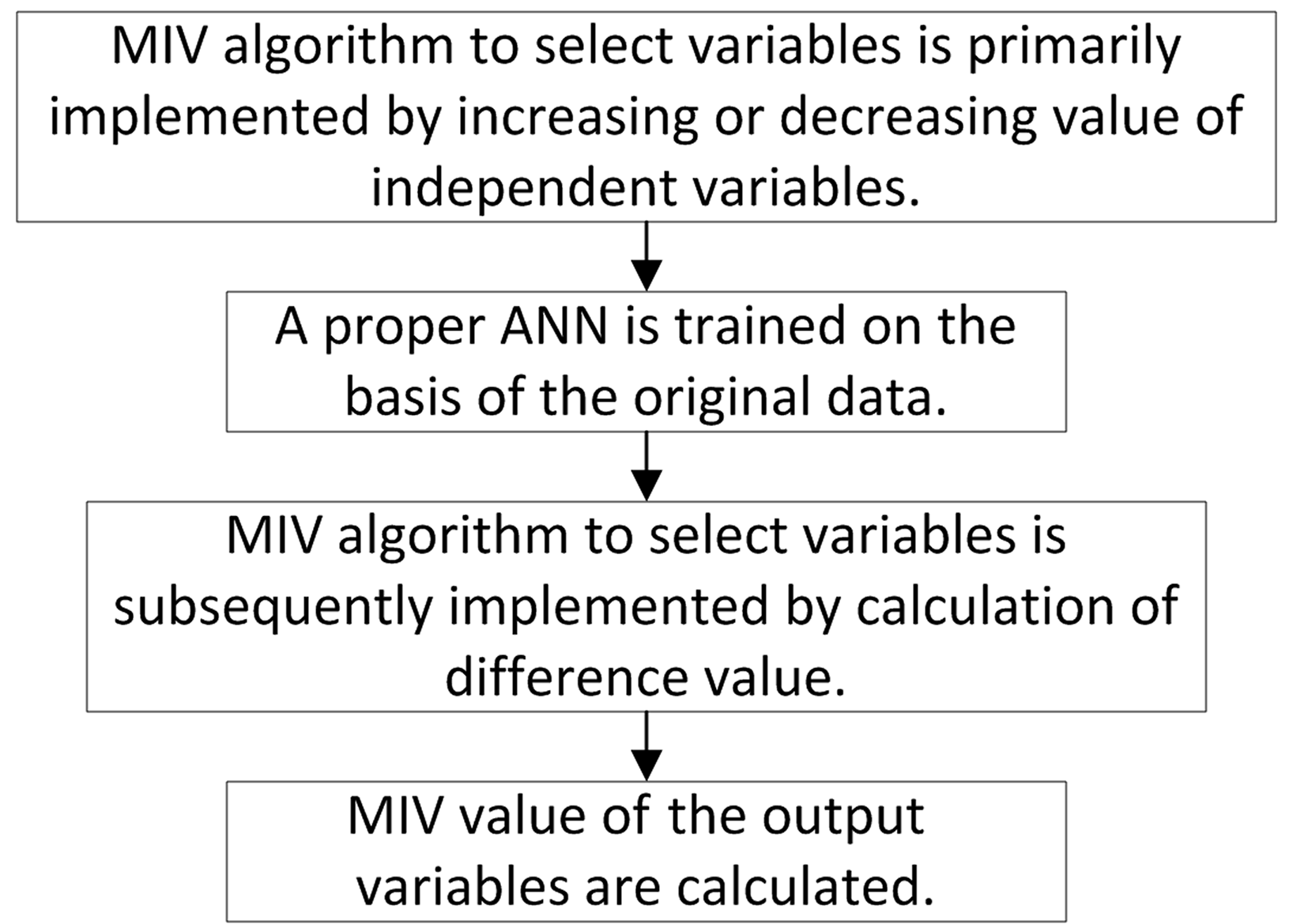

Supplement: Figure S2 — The flowchart to select variables on the basis of MIV. (TIF) [file pntd.0002123.s005.tif]

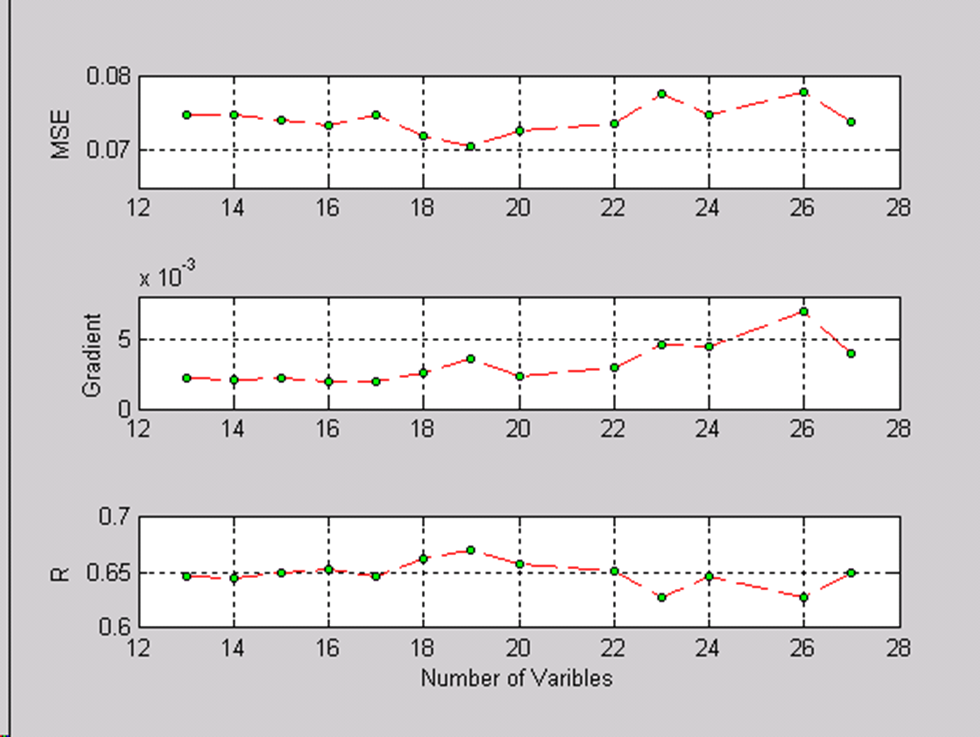

Supplement: Figure S3 — The plot showing the performance of network which containing different number of risk factors. (TIF) [file pntd.0002123.s006.tif]
