# Supplementary material for: Transmission Risks of Schistosomiasis Japonica: Extraction from Back-propagation Artificial Neural Network and Logistic Regression Model
Source: PLoS Negl Trop Dis. 2013 Mar 21;7(3):e2123. doi: 10.1371/journal.pntd.0002123 (PMC3605232; doi:10.1371/journal.pntd.0002123)
Supplement: Text S1 — The procedure of screening risk factors. (DOC) [file pntd.0002123.s007.doc]

**Supplementary Text S1**

**The procedure of screening risk factors**

In this study, we developed a new way to select the input variables in order to get the optimal network, which was integrated with three values, e.g. mean impact value (MIV), the magnitude of gradient, and R. As a result, we found three networks was better fitted according to the above four evaluating indices, namely the networks A, B, C, containing 19, 18, 16 input variables, respectively. Generally, the input variables of network are selected on the basis of professional knowledge and experience, since the precision of network will be lowered if network contain some variables which are less significant. In this study, we screened the input variables based on the performance of BP artificial neural networks and MIV of variables in the networks. A proper neural network was synthetically evaluated by the following four indices, e.g. MSE, the magnitude of gradient, validation checks, and correlation coefficient R. Then, we defined that the network with 16 input variables was the optimal network (1) MIV reflects the change of weight matrix in the network, which is considered one of the best indices to evaluate relationship between variables. Principally, the variable with the smallest MIV would be removed from the network until there is no very small MIV in the network. (2) In the study, when the network containing 25 input variables, it didn’t remain stable because there was pulsation in the procedure of training. So that we removed two variables with the smallest MIV in the network employed 26 input variables. (3) The network containing 21 input variables was analogous with the network with 25 input variables. For the network with 19 input variables, MSE and R were better than any other networks, and the magnitude was also not poor. But there were the variable with small MIV. (4) The performance of the network containing 16 input variables was acceptable according to its four evaluation indices. (5) Although there also were no variable with very small MIV in the networks containing 15, 14, 13 input variables, respectively, all these three networks were worse than those values, e.g. MSE, the magnitude of gradient and R, of the network when employed with 16 input variables.
